# Mek1/Mre4 is a master regulator of meiotic recombination in budding yeast

**DOI:** 10.15698/mic2016.03.487

**Published:** 2016-02-22

**Authors:** Nancy M. Hollingsworth

**Affiliations:** 1 Department of Biochemistry and Cell Biology, Stony Brook University, Stony Brook, NY 11794-5215.

**Keywords:** Mek1, kinase, Zip1, meiosis, recombination, synaptonemal complex, phosphorylation, SILAC, Cdc7-Dbf4, DDK

## Abstract

Sexually reproducing organisms create gametes with half the somatic cell chromosome number so that fusion of gametes at fertilization does not change the ploidy of the cell. This reduction in chromosome number occurs by the specialized cell division of meiosis in which two rounds of chromosome segregation follow a single round of chromosome duplication. Meiotic crossovers formed between the non-sister chromatids of homologous chromosomes, combined with sister chromatid cohesion, physically connect homologs, thereby allowing proper segregation at the first meiotic division. Meiotic recombination is initiated by programmed double strand breaks (DSBs) whose repair is highly regulated such that (1) there is a bias for recombination with homologs rather than sister chromatids, (2) crossovers are distributed throughout the genome by a process called interference, (3) crossover homeostasis regulates the balance between crossover and non-crossover repair to maintain a critical number of crossovers and (4) each pair of homologs receives at least one crossover. It was previously known that the imposition of interhomolog bias in budding yeast requires meiosis-specific modifications to the DNA damage response and the local activation of the meiosis-specific Mek1/Mre4 (hereafter Mek1) kinase at DSBs. However, because inactivation of Mek1 results in intersister, rather than interhomolog DSB repair, whether Mek1 had a role in interhomolog pathway choice was unknown. A recent study by Chen *et al*. (2015) reveals that Mek1 indirectly regulates the crossover/non-crossover decision between homologs as well as genetic interference. It does this by enabling phosphorylation of Zip1, the meiosis-specific transverse filament protein of the synaptonemal complex (SC), by the conserved cell cycle kinase, Cdc7-Dbf4 (DDK). These results suggest that Mek1 is a “master regulator” of meiotic recombination in budding yeast.

Chen *et al*. (2015) applied an unbiased phosphoproteomic approach to detect potential Mek1 substrates. Stable Isotope Labeling of Amino Acids in Cell Culture (SILAC) was used to compare the levels of different phosphopeptides with or without Mek1 kinase activity in cells arrested with unrepaired DSBs. Peptides containing Mek1 phosphorylation sites should be underrepresented in the culture in which Mek1 was inhibited. By this assay, three out of four adjacent serines in the C-terminus of Zip1 were potentially regulated by Mek1. Zip1 contains two globular domains at the N and C termini that flank a coiled coil region that promotes homo-oligomerization. The C terminal domain interacts with the axial elements formed by condensation of sister chromatids to form the tripartite SC structure. The serines phosphorylated in Zip1 (designated 4S) are contained within a part of the C-terminal domain required for synapsis and recombination. Although transverse filament proteins of the SC are poorly conserved at the primary sequence level, the Zip1 4S region shares homology with transverse filament proteins from zebrafish and mammals.

In addition to “gluing” homologous pairs of sister chromatids together in the SC, Zip1 is a member of the functionally diverse set of “Zmm” proteins that promotes the formation of recombination intermediates that are preferentially resolved as interfering crossovers. Other labs have shown an antagonistic relationship between the Sgs1-Top3-Rmi1 (STR) complex and the Zmms such that Zmm proteins stabilize recombination intermediates, protecting them from disassembly by STR. These intermediates go on to become double Holliday junctions that are preferentially resolved as crossovers that are distributed throughout the genome by interference. In the absence of *ZMM* function, non-crossover formation is increased by STR-promoted synthesis-dependent strand annealing (SDSA). In addition, double Holliday junction intermediates are resolved in an unbiased manner by structure-specific nucleases (SSNs) such as Mus81-Mms4 to produce both crossover and non-crossover products.

Phenotypic analysis of mutants in which the four serines were substituted with alanine (to prevent phosphorylation), *zip1-4A*, or with aspartic acid (to mimic phosphorylation), *zip1-4D*, revealed that phosphorylation of the 4S region is necessary for the *ZMM* function of Zip1. While *zip1-4D* phenotypes resembled *ZIP1*, the *zip1-4A* phenotypes were either equivalent or more severe than *zip1*∆. *ZMM* genes like *ZIP1* are required for a type of interhomolog engagement that downregulates DSB formation, especially on larger chromosomes. Physical analysis of recombination at a DSB hotspot on chromosome III indicated a delay in the disappearance of DSBs in *zip1-4A* and an accumulation to levels even greater than the *zip1*∆. This observation was confirmed by an assay that monitors total DSB levels as well. Non-crossovers at the hotspot were increased in *zip1-4A* in an *SGS1*-dependent manner, while crossovers were reduced. In addition, the *zip1-4A* mutant exhibited delays in the transition from DSBs to joint molecule intermediates. Taken together, these results show that Zip1-4S phosphorylation, like the *ZMM*s, is necessary to protect recombination intermediates from the action of Sgs1, thereby allowing them to mature into crossovers. Zip1 4S phosphorylation therefore provides a mechanism by which the crossover/non-crossover decision can be regulated to mediate crossover homeostasis. For example, as DSB levels decrease, maintaining the number of breaks associated with phosphorylated Zip1 could keep the number of crossovers constant.

Genetic analysis of recombination on both the small chromosome III and the large chromosome VII yielded an important finding. In *zip1-4A*, crossovers in three different intervals on Chromosome III were reduced, consistent with the physical analysis of the chromosome III hotspot, but on chromosome VII, the map distances of three intervals were increased over wild type. Interference was reduced or abolished on both chromosomes, however, suggesting that the *ZMM* pathway was eliminated throughout the genome by failure to phosphorylate Zip1. The hypothesis is that a disproportionate number of DSBs occurring on the large chromosome due to a failure in interhomolog engagement creates more opportunities for crossovers to occur by an alternative pathway that utilizes SSNs such as Mus81-Mms4. Consistent with this idea, deletion of *MUS81* reduced the spore viability of *zip1-4A* to the same level as *zip1*∆ *mus81*∆.

Combining *zip1-4A* with a meiotic depletion allele of *SGS1*, *sgs1-md*, produced another surprising result—the cells arrested in meiotic prophase. This arrest was relieved by preventing DSB formation, indicating that unrepaired breaks in *sgs1-md zip1-4A* diploids trigger the meiotic recombination checkpoint. Importantly, this is a gain of function phenotype as *zip1*∆ *sgs1-md* mutants do not arrest. Epistasis analysis between *sgs1-md zip1-4A* and various *ZMM* deletions demonstrated that Zip1 phosphorylation functions prior to the other *ZMM*s. This result is consistent with chromatin immunoprecipitation experiments by the Borde lab showing that *ZIP1* is required for the recruitment of the Zmm protein, Zip3, to DSBs. Chen *et al*. (2015) propose that Zip1 has two temporally separate functions. First, Zip1 is recruited to DSBs and phosphorylation of Zip1 4S commits those breaks to be repaired by the *ZMM* pathway. Second, after stable interhomolog connections have been made, polymerization of Zip1 results in SC formation. In fact, chromosomes in *zip1-4A* exhibit Zip1-4A foci, but very little synapsis. The authors propose that DSBs remain unrepaired unless the Zip1-4A protein is removed by Sgs1, in which case the breaks are repaired either by SDSA or the SSN pathway.

The dependence of Zip1 4S phosphorylation on Mek1 kinase activity was confirmed using a phosphospecific antibody to Zip1 S816, validating the SILAC results. However, Mek1 did not phosphorylate recombinant Zip1 *in vitro*. This result, as well as the fact that the Zip1 4S sequence does not match the Mek1 phosphorylation consensus (RXXT), indicates that regulation of Zip1 4S phosphorylation by Mek1 is indirect. DDK is a highly conserved cell cycle kinase required for the initiation of DNA replication. In meiosis, DDK is required for meiotic DSB formation, the mono-orientation of sister kinetochores at Meiosis I and the induction of the meiosis-specific transcription factor, *NDT80*, which is necessary for Holliday junction resolution, SC disassembly and meiotic progression. DDK directly phosphorylated Zip1 S816 *in vitro* and promoted S816 phosphorylation *in vivo* when the Zip1 C terminal domain was ectopically expressed in vegetative yeast cells. Chen *et al*. (2015) propose that Mek1 kinase activity enables DDK to phosphorylate Zip1 4S, either by creating a “landing pad” for DDK, or by making DSBs accessible to DDK. For example, Mek1 has been proposed to locally counteract cohesion to allow release of the DSB ends from the axis.

**Figure 1:**
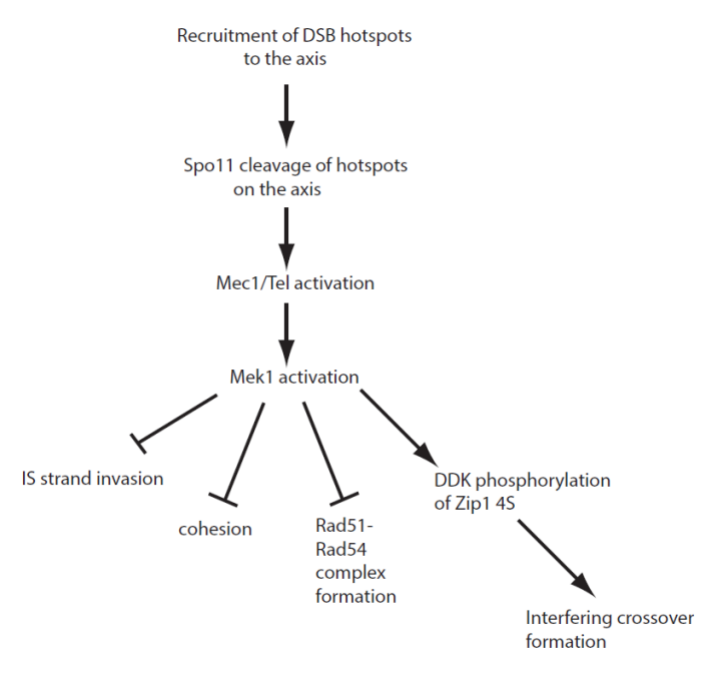
FIGURE 1: Activation of Mek1 activity at meiotic DSBs globally regulates different aspects of recombination. DSBs occur in preferred regions of the genome called “hotspots” and are created by Spo11 cleavage after the hotspot regions have been tethered to chromosome axes. DSB formation and resection activate the Tel1 and Mec1 checkpoint kinases, respectively, which in turn activate the meiosis-specific kinase Mek1 locally at DSBs. Mek1 kinase activity then represses intersister strand invasion by phosphorylation of unknown substrates. It is proposed to locally antagonize cohesion, although the mechanism for this remains to be determined. Mek1 phosphorylation of Rad54 decreases Rad51-Rad54 complex formation, helping to maintain Rad51 in an inactive state while Dmc1 mediates interhomolog recombination. In addition, Mek1 allows DDK phosphorylation of Zip1 4S which is required for interhomolog engagement, chromosome synapsis, and the interfering crossover pathway of recombination.

In summary, the local activation of Mek1 at meiotic DSBs not only imposes interhomolog bias, but also indirectly controls the formation of interfering crossovers and provides a mechanism for mediating crossover homeostasis. Mek1 is therefore in the position to coordinate these different regulatory processes (Figure 1). Determining the proteins whose phosphorylation is dependent on Mek1, either directly or indirectly, will be key to elucidate the mechanisms by which meiotic recombination is regulated in yeast. Although a mammalian Mek1 ortholog has not yet been identified, the conservation of both the Zip1 4S region and DDK in mammals raises the possibility that regulation of recombination through phosphorylation of this region is similarly conserved.

